# Diagnostic value of non-magnifying gastroscopy followed by targeted biopsy for detection of early gastric cancer: Multicenter prospective study

**DOI:** 10.1055/a-2781-6191

**Published:** 2026-03-16

**Authors:** Fumei Yin, Qinwei Xu, Lianjun Di, Xin Wang, Lang Yang, Shan Hu, Mingjie Zhang, Yilin Wang, Heng Zhang, Changwei Duan, Jianqiu Sheng, Rui Xie, Xiao Hu, Peng Jin

**Affiliations:** 1 651943 Senior Department of Gastroenterology, Chinese PLA General Hospital First Medical Center, Beijing, China; 2 74639 Department of Gastroenterology, Beijing Chao-Yang Hospital Capital Medical University, Beijing, China; 3 66324 Department of Gastroenterology, Shanghai East Hospital, Shanghai, China; 4 159358 Department of Gastroenterology, Affiliated Hospital of Zunyi Medical University, Zunyi, China; 5 Department of Gastroenterology, Chinese PLA General Hospital Seventh Medical Center, Beijing, China; 6 89669 Department of Gastroenterology and Hepatology, University of Electronic Science and Technology of China Sichuan Provincial People's Hospital, Chengdu, China; 7 12599 Medical School of Chinese PLA, Beijing, China

**Keywords:** Endoscopy Upper GI Tract, Precancerous conditions & cancerous lesions (displasia and cancer) stomach, Diagnosis and imaging (inc chromoendoscopy, NBI, iSCAN, FICE, CLE), Quality and logistical aspects, Performance and complications

## Abstract

**Background and study aims:**

Non-magnifying endoscopy remains the most essential tool for detecting early gastric cancer (EGC), but there is still a lack of widely accepted diagnostic methods. We established an optimized EGC detection protocol using non-magnifying gastroscopy-guided targeted biopsy of morphological suspicious lesions and evaluated its effectiveness.

**Methods:**

This study included 5738 participants across four medical centers in China. Targeted biopsies were performed on the following suspicious lesions for high-grade neoplasia (HGN) under screening non-magnifying gastroscopy: 1) ulcerative lesions; 2) esophagogastric junction reddish lesions outside the atrophic area; 3) pale or well-demarcated lesions outside the atrophic area, except polypoid lesions smaller than 5 mm; 4) elevated lesions with clear borders or uneven top within the atrophic area; and 5) flat/depressed lesions with irregular borders or uneven surface or ocher color under narrow-band imaging (NBI) within the atrophic area. Sensitivity for detecting gastric HGN and the positive predictive value (PPV) of targeted biopsy were calculated.

**Results:**

The targeted biopsy method demonstrated a sensitivity of 90.9% (50/55, 95% confidence interval [CI] 83.1%-98.8%) and a PPV of 5.4% (50/931, 95% CI 3.9%-6.8%) for diagnosing HGN on per-lesion assessment. Lesions that met the suspicious morphological criteria carried a significant risk to be HGNs, even after adjusting for age, sex, and other risk factors associated with gastric cancer (adjusted odds ratio = 42.03, 95% CI 11.14–158.63, *P* < 0.001).

**Conclusions:**

Targeted biopsy of suspicious lesions for HGN with non-magnifying gastroscopy can be used as a primary clue for detecting EGC.

## Introduction

1

Gastric cancer is one of the most common malignant tumors worldwide, with particularly high incidence in East Asia, Eastern Europe, and South America. The prognosis for gastric cancer is generally poor, but early detection could significantly reduce mortality and improve patient quality of life. The diagnostic gold standard for early gastric cancer (EGC) is gastroscopy combined with histological confirmation through biopsy. Currently, clinical practice utilizes two endoscopic approaches: non-magnifying and magnifying gastroscopy. Comparative studies demonstrate magnifying gastroscopy’s superior diagnostic accuracy over conventional non-magnifying gastroscopy, particularly for small depressed mucosal carcinomas 1 . Thus, the Japan Gastroenterological Endoscopy Society recommended magnifying endoscopy with the vessel-plus-surface classification system for differential diagnosis of cancer and non-cancerous lesions 2 . However, magnifying endoscopy is mainly used for characterization but not for detection of lesions suspected to be EGC. Non-magnifying gastroscopy still serves as the foundational diagnostic tool and first-line screening modality for detecting EGC.

However, conventional non-magnifying gastroscopy demonstrates limited efficacy for detecting EGC when deployed without standardized diagnostic method, as evidenced by an Asian screening program 3 . Previously, some basic principles of EGC screening using non-magnifying gastroscopy were proposed. Yao et al. reported that endoscopic well-demarcated border and irregularity in color/surface pattern inferred diagnosis of EGC 4 . Gotoda et al. suggested that changes in mucosal color and height, loss of the background vascular network, and spontaneous bleeding were indicative of cancerous lesions under non-magnifying gastroscopy, as well as that the morphological type and color of EGC correlates with its histology 5 . For example, the differentiated type of flat/depressed lesions appear reddish, whereas the undifferentiated type appears pale or faded. In addition, the location of EGC varies according to its histological types. Undifferentiated types frequently occur at the boundary of the atrophic region or in adjacent areas, whereas well-differentiated types are more common within the atrophic area 6 . Despite these findings, non-magnifying gastroscopy lacks widely accepted diagnostic criteria supported by multicenter validation studies.

**Fig. 1:**
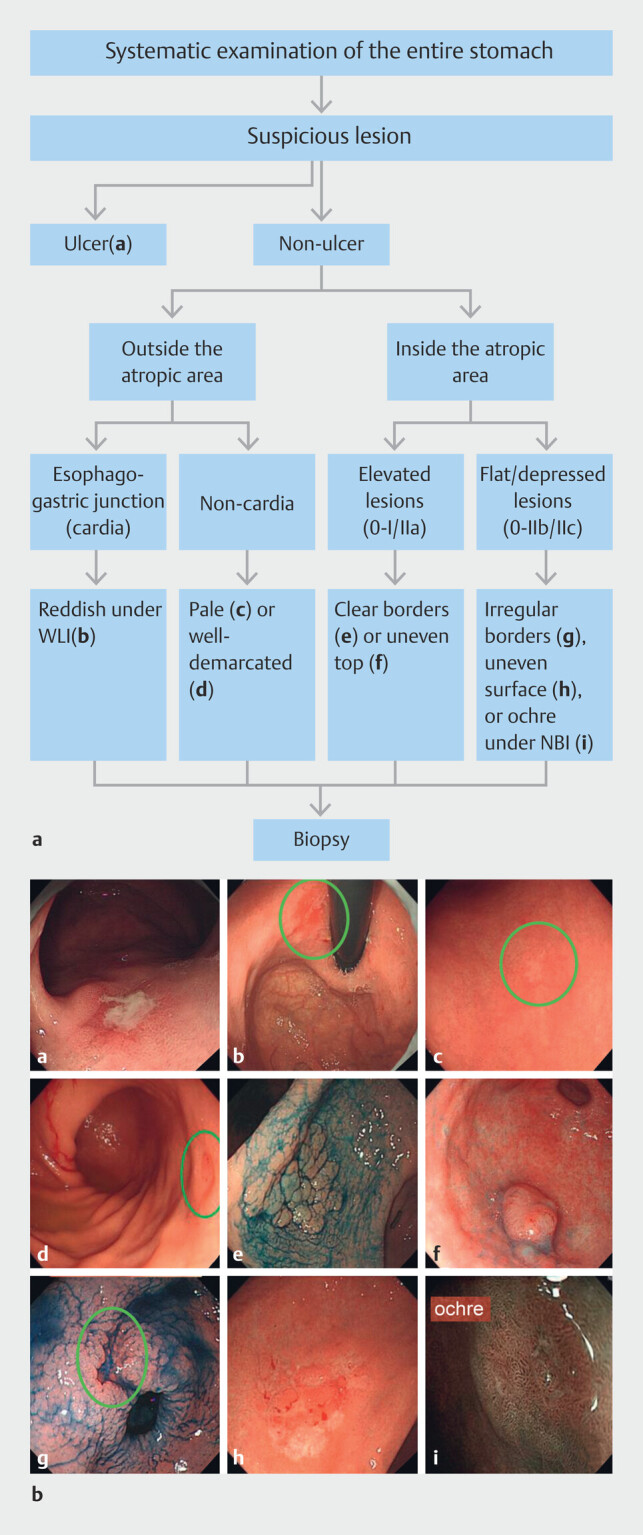
Suspicious lesions under non-magnifying gastroscopy. Indigo carmine spraying was utilized in **e** and **g** to delineate borders of the lesions.

Based on the previously described principles and through our retrospective analysis of 890 cases of EGC and gastric dysplasia 7 , we developed an enhanced protocol for detecting EGC by targeted biopsy of morphologically suspicious lesions for high-grade neoplasia (HGN) with non-magnifying gastroscopy. Suspicious lesions for HGN include the following types of lesions as shown in Fig. 1 : 1) ulcerative lesions; 2) esophagogastric junction (cardia) 8 reddish lesions outside the atrophic area; 3) pale or well-demarcated lesions outside the atrophic area, except polypoid lesions smaller than 5 mm; 4) elevated lesions with clear borders or uneven top within the atrophic area; and 5) flat/depressed lesions with irregular borders or uneven surface or ochre color under narrow-band imaging (NBI) within the atrophic area. Recently, we validated the efficacy of this protocol through a single-center prospective study and demonstrated sensitivity of 83.3% for detecting HGN 9 . Previous studies indicated that sensitivity of magnifying NBI for screening EGC was approximately 60% 1 10 . Therefore, we hypothesized that this method of targeted biopsy under non-magnifying gastroscopy could be effective for diagnosing EGC.

We conducted a large-scale, multicenter, cross-sectional diagnostic study to evaluate the performance of this method. The primary objective of this study was to investigate whether it detects most of HGNs.

## Patients and methods

### Study design and participants

From February 2022 to August 2022, this study was conducted at four tertiary hospitals in China including the Seventh Medical Center of Chinese PLA General Hospital, Sichuan Provincial People’s Hospital, Shanghai East Hospital, and Affiliated Hospital of Zunyi Medical University and registered on the Chinese Clinical Trial Registry System (No. ChiCTR2200057062). The study was conducted in accordance with the Declaration of Helsinki and received approval from the ethics committee of each participating hospital. The findings were reported following the STARD (Standards for Reporting Diagnostic Accuracy) 2015 statement 11 . All eligible participants provided written informed consent before participation.

This study included consecutive participants aged 45 to 74 years old who visited each medical center for routine esophagogastroduodenoscopy screening and agreed to participate in the study. Exclusion criteria included individuals with known untreated gastric cancer or precancerous lesions or other upper gastrointestinal malignancies, those undergoing therapeutic or emergency gastroscopy, those who had undergone partial or total removal of the stomach, patients with severe conditions such as heart or respiratory failure that could not go through close observation under gastroscopy, patients with conditions such as gastric retention or esophageal stricture that prevent complete observation of the stomach, and those with obvious advanced gastric cancer such as invasive mass or ulcer under gastroscopy.

After signed informed consents, all eligible subjects completed a questionnaire with the help of trained study staff. The questionnaire collected baseline information that has previously been reported to be related to gastric cancer risk 12 , including age, sex, lifestyle factors (smoking and dietary habits such as consumption of pickled or fried food), history of *H. pylori* infection and eradication, history of diseases associated with increased risk of gastric adenocarcinoma (gastric atrophy, intestinal metaplasia, autoimmune atrophic gastritis, endoscopically resected gastric epithelial dysplasia or early gastric adenocarcinoma, or a history of any type of cancer), and family history of gastric cancer among first-degree relatives. Then gastroscopy was scheduled for all participants.

### Procedure

The high-definition endoscope used in this study was a GIF H260, HQ290, or H290 with a CV-290 video processor (Olympus Corporation, Tokyo, Japan). According to the recommendation of the Chinese Society of Digestive Endoscopy 13 , pronase and simethicone were administered orally 10 minutes before gastroscopy. A comprehensive examination of the entire stomach was conducted and a minimum of 40 images of the stomach should be taken. Each examination took no less than 7 minutes 13 .

All endoscopists participating in this study were well-trained experts (≥ 5 years of endoscopic experience and who had performed gastroscopy in over 5000 cases) in endoscopic screening and diagnosis of early gastrointestinal neoplasm and received training on the research protocol before the study began. Endoscopic findings were recorded by participating endoscopists at the time of the procedure and subsequently reviewed by a central assessment team (four senior experts with ≥ 10 years endoscopic experience) to ensure consistency. The primary analyses were based on the centrally adjudicated findings.

During the gastroscopy procedure, first the extent of the atrophic border was determined under white light imaging (WLI) and NBI. Endoscopic features of atrophy included paler areas, loss of folds, and prominence of vessels 14 15 . Then the endoscopists carefully looked for distinctive lesions inside and outside the atrophic area and biopsies were taken of any lesions that met the previously described “suspicious” morphological characteristics as shown in Fig. 1. In addition, endoscopists performed biopsies on any lesions or sites that do not the previously described morphological characteristics, but could not be excluded as neoplasms based on personal experience, or just random biopsy. Indigo carmine spraying and NBI could be utilized to delineate borders and surfaces of the lesions.

All biopsied lesions and corresponding histology were recorded in the case report forms, as well as use of sedation or not, extent of gastric atrophy (non-atrophy, distal atrophy i.e. affecting antrum or incisura, proximal atrophy i.e. affecting the corpus with or without the antrum and incisura) 16 , intestinal metaplasia, and presence of obvious active gastritis or not.

### Pathological evaluation

Pathological diagnoses were made on biopsied tissue or resected specimens obtained by endoscopic or surgical removal. Each pathological slide was reviewed by two senior pathologists. If biopsied tissues and resected specimens were both available, the latter was used for final diagnosis. Pathological results were classified according to the revised Vienna classification 17 : category 1 (negative for neoplasia, C1), category 2 (indefinite for neoplasia, C2), category 3 (mucosal low-grade neoplasia, C3), category 4 (mucosal HGN, C4), and category 5 (submucosal invasion by carcinoma, C5). Categories 3, 4, and 5 comprised definitive gastric epithelial neoplasia (GEN), whereas categories 4 and 5 represented HGN. Non-epithelial neoplasia such as lymphoma and gastrointestinal stromal tumor, as well as neuroendocrine tumors, were categorized as category X (Cx) in our study.

### Outcomes and sample size

The primary outcome was sensitivity for detecting gastric HGN, which was calculated as the proportion of HGNs detected by targeted biopsy of aforementioned suspicious lesions among all HGNs detected during gastroscopy screening. The secondary outcome was the positive predictive value (PPV) of targeted biopsy, which was calculated by the proportion of HGNs among all targeted biopsied suspicious lesions.

Our previous studies showed that this protocol detected 83.3% to approximately 90.5% of gastric HGNs on screening gastroscopy 9 18 . Based on other two prospective studies, magnifying gastroscopy had a sensitivity of about 60% in screening for EGC, and non-magnifying gastroscopy showed no significant difference in the epithelial neoplasm detection rate compared with magnifying gastroscopy 1 10 . The study was designed to have a power of 90% to test the hypothesis that targeted biopsy of suspicious lesions would have a sensitivity of at least 60% for detection of gastric HGNs under the null hypothesis, with a significance level of 0.05 (1-sided) and the alternative hypothesis of 80% for sensitivity. The detection rate for HGNs among patients aged 45 to 74 years undergoing gastroscopy in 2021 at the four participating centers was calculated as four cases per 1,000 population. Thus, at least 45 HGNs or 11,250 enrolled participants were required to achieve the primary objective. PASS 11 software (NCSS, LLC., Utah, United States) was used to calculate the sample size.

In August 2022, 6 months after the beginning of enrollment, a total of 5738 participants were assessed and 55 HGNs had been detected, surpassing the minimum number of HGNs (45 cases) required by the study design. Therefore, all authors agreed to terminate recruitment before reaching the initially expected number of participants. The revised sample size was sufficient to achieve the predetermined 90% power with one-sided alpha of 0.05.

### Statistical analysis

Count data were compared using chi-square test or Fisher's exact test. Comparisons of continuous data were performed using the *t* test. Adjusted odds ratios were calculated through multivariate logistic regression analysis, with presence of HGN as the dependent variable and the following covariates: sex, age, *H. pylori* infection status, smoking, consumption of pickled food, fried food, personal history of gastric neoplasia, malignancies other than gastric cancer, family history of gastric cancer in first-degree relatives, active gastritis, intestinal metaplasia, distal/proximal atrophic gastritis, and morphology of lesions conforming to any types shown in Fig. 1 . Missing values were addressed using the multiple imputation method. Statistical analysis was performed using SPSS version 26 software (IBM Corp, Armonk, New York, United States). A statistically significant difference was established when *P* < 0.05.

## Results

### Characteristics of participants

**Fig. 2:**
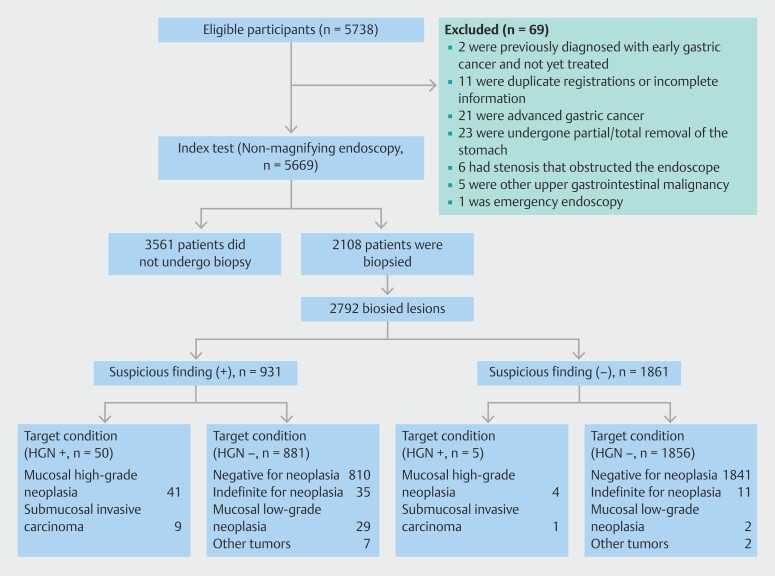
Flowchart of participants and outcomes.

Between February 2022 and August 2022, 5738 participants were assessed for study eligibility and 69 were excluded for various reasons ( Fig. 2 ). Finally, 5669 participants were included in the primary analysis, comprising 2668 men and 3001 women. The average age of participants was 58.2 ± 7.7 years (mean ± SD). Baseline data for these participants are shown in Table 1 .

**Table:** **Table 1** Baseline data for study participants (n = 5669).

General condition	Participants, n (%)
Sex	
Male	2668 (47.1)
Female	3001 (52.9)
Age (years)	
45–59	3403 (60.0)
60–74	2266 (40.0)
*H. pylori* infection	
Never infected	1891 (33.3)
Previous/present infected	1479 (26.1)
Unknown	2299 (40.6)
Mucosal atrophy	
Non-atrophic	2860 (50.4)
Distal gastric atrophy*	1700 (30.0)
Proximal gastric atrophy*	990 (17.5)
Unknown	60 (1.0)
History of gastric neoplasms ^†^	
No	5575 (98.3)
Yes	94 (1.7)
Unknown	2 (0.0)
Malignant tumors other than gastric cancer	
No	5103 (90.0)
Yes	162 (2.9)
Unknown	404 (7.1)
History of gastric cancer in first degree relatives	
No	3903 (68.8)
Yes	469 (8.3)
Unknown	1297 (22.9)
Smoking	
No	4436 (78.3)
Yes	818 (14.4)
Unknown	415 (7.3)
Pickled food	
No	5070 (89.5)
Yes	183 (3.2)
Unknown	416 (7.3)
Fried food	
No	5168 (91.2)
Yes	85 (1.5)
Unknown	416 (7.3)
*Distal atrophy = antral and antral predominant (Kimura-Takemoto classification C-1, C-2); Proximal atrophy = corpus predominant (C-3, O-1, O-2), pan-atrophy (O-3) and autoimmune atrophic gastritis. ^†^ Gastric neoplasms included gastric cancers and dysplasia.	

Among these individuals, 3561 showed no signs of suspicious neoplastic lesions during gastroscopy and no biopsy was performed on them. The remaining 2108 subjects underwent biopsy of 2792 gastric lesions during gastroscopy, including 931 lesions that had the “suspicious” morphological characteristics, as shown in Fig. 1 , and the other 1861 lesions did not meet the above morphological characteristics, but could not be excluded as neoplasms based on endoscopist personal experience or just random biopsy ( Fig. 2 ).

### Diagnostic performance

A total of 55 gastric HGNs and submucosal invasive carcinomas from 52 patients were detected among these 5669 participants. Endoscopic and histological features of the 55 HGNs are summarized in Table 2 . Among them, 50 HGNs were detected by targeted biopsy of suspicious morphological characteristics, with a sensitivity of 90.9% (50/55, 95% confidence interval [CI] 83.1%-98.8%). The PPV of targeted biopsy was 5.4% (50/931, 95% CI 3.9%-6.8%), which indicated that approximately 19 targeted biopsies could identify an HGN. For all 86 GENs (all definitive GEN) detected during gastroscopy, targeted biopsy identified 79 GENs with a sensitivity of 91.9% (79/86, 95% CI 86.0%-97.8%) and a PPV of 8.5% (79/931, 95% CI 6.7%-10.3%). Among 1861 lesions biopsied that did not meet suspicious morphological characteristics, only five HGNs (0.3%, 95% CI 0.0%-0.5%) were confirmed by pathology, whereas 98.9% of these lesions (1841/1861, 95% CI 98.5%-99.4%) were pathologically negative (negative predictive value [NPV]) for neoplasia ( Table 3 ).

**Table:** **Table 2** Endoscopic and histological features of 55 HGNs **.**

	Biopsied based on suspicious morphological characteristics (n = 50)	Biopsied based on other experience or randomly (n = 5)
HGN location, n (%)		
Upper third	9 (18.0)	2 (40.0)
Middle third	11 (22.0)	1 (20.0)
Lower third	30 (60.0)	2 (40.0)
Tumor macroscopic type, n (%)		
Ulcer	11 (22.0)	0 (0.0)
Elevated (0-I/IIa)	12 (24.0)	1 (20.0)
Flat/ depressed (0-IIb/IIc)	27 (54.0)	4 (80.0)
Tumor size, n (%)		
≤ 5 mm	6 (12.0)	0 (0.0)
5–10 mm	10 (20.0)	2 (40.0)
≥ 10 mm	34 (68.0)	3 (60.0)
Tumor depth, n (%)		
Mucosa	41 (82.0)	4 (80.0)
Submucosa	9 (18.0)	1 (20.0)
Pathology, n (%)		
Well-moderately differentiated	41 (82.0)	4 (80.0)
Poorly-differentiated	9 (18.0)	1 (20.0)
HGN, high-grade neoplasia.		

**Table:** **Table 3** Diagnostic performance of non-magnifying gastroscopy with targeted biopsy.

	HGN (n, 95% CI)	GEN (n, 95% CI)
Sensitivity	90.9% (50/55, 83.1%–98.8%)	91.9% (79/86, 86.0%–97.8%)
Specificity	67.8% (1856/2737, 66.1%–69.6%)	68.5% (1854/2706, 66.8%–70.3%)
Accuracy	68.3% (1906/2792, 66.5%–70.0%)	69.2% (1933/2792, 67.5%–70.9%)
PPV	5.4% (50/931, 3.9%–6.8%)	8.5% (79/931, 6.7%–10.3%)
NPV		98.9% (1841/1861, 98.5%–99.4%)
CI, confidence interval; GEN, gastric epithelial neoplasia; HGN, high-grade neoplasia; NPV, negative predictive value; PPV, positive predictive value.		

Lesions that morphological characteristics, as shown in Fig. 1 , carried a significant risk of HGN, even after adjustment for age, sex, and other risk factors for gastric cancer (adjusted OR 42.03, 95% CI 11.14–158.63, *P* < 0.001, Table 4 ). Therefore, lesions with these morphological features were indeed suspicious for HGN. Proximal atrophy was another independent risk factor for HGN (adjusted OR 32.65, *P* = 0.003).

**Table:** **Table 4** Univariate and multivariate logistic regression analysis of risk factors for HGN (n = 5661).

Factors	Reference	Risk Ratio (95%CI)	*P* value	Adjusted odds ratio (95%CI)	*P* value
Sex	Male	0.43 (0.24–0.77)	0.004	0.85 (0.30–2.39)	0.766
Age (years)	45–59	2.06 (1.19–3.59)	0.01	0.65 (0.25–1.73)	0.386
*H. pylori* infection	No	2.35 (1.12–4.91)	0.024	0.87 (0.31–2.45)	0.788
Smoking	No	1.72 (0.90–3.31)	0.103	2.00 (0.67–5.54)	0.210
Pickled food	No	4.64 (2.06–10.47)	< 0.001	2.84 (0.80–10.01)	0.107
Fried food	No	3.98 (1.21–13.05)	0.023	0.98 0.10–9.39)	0.985
History of gastric neoplasm*	No	3.79 (1.16–12.39)	0.028	1.49 (0.26–8.59)	0.659
Malignant tumors other than gastric cancer	No	2.03 (0.62–6.58)	0.240	5.20 (0.54–50.33)	0.155
History of gastric cancer in first degree relatives	No	1.73 (0.72–4.20)	0.22	1.74 (0.53–5.73)	0.364
Active gastritis	No	2.00 (1.07–3.72)	0.03	1.26 (0.43–3.70)	0.679
Intestinal metaplasia	No	4.73 (1.99–11.27)	< 0.001	0.34 (0.07–1.67)	0.182
Mucosal atrophy			< 0.001	–	< 0.001
Distal ^†^	Non-atrophy	7.33 (2.09–25.77)	0.002	5.97 (0.58–61.56)	0.133
Proximal ^†^	Non-atrophy	31.70 (9.69–103.75)	< 0.001	32.65 (3.37–316.26)	0.003
Suspicious morphological characteristics	No	82.67 (29.72–229.95)	< 0.001	42.03 (11.14–158.63)	< 0.001
CI, confidence interval; OR, odds ratio. *Gastric neoplasms included gastric cancers and dysplasia. ^†^ Distal atrophy is antral and antral predominant (Kimura-Takemoto classification C-1, C-2); proximal atrophy is corpus predominant (C-3, O-1, O-2), pan-atrophy (O-3) and autoimmune atrophic gastritis.					

Among the detected HGNs, the most common type were flat or depressed lesions within the atrophic area (36.3%, 20/55), followed by ulcerative lesions (20.0%, 11/55) (Supplementary Fig. 1). For the PPV of targeted biopsy, elevated lesions with uneven tops were most likely to be HGN (9.4%, 5/53), followed by ulcerative lesions (8.9%, 11/123) (Supplementary Table 1).

## Discussion

Endoscopic screening could reduce gastric cancer mortality in Asian countries 19 . However, detecting gastric dysplasia and EGC poses a significant challenge due to the absence of well-defined endoscopic features under non-magnifying gastroscopy 16 . Although it has been noted that EGC often presents with a brownish color surrounded by green epithelium under NBI 20 , a recent randomized controlled trial showed that NBI 21 with or without magnifying endoscopy did not increase the detection rate for EGC over conventional WLI 21 22 . Non-magnifying gastroscopy remains the most essential tool for detecting EGC. To date, there are few widely accepted diagnostic methods. Existing approaches are generally based on personal experience of experts and are lacking in robust clinical evidence 4 5 23 . In this study, we introduced a method of targeted biopsy of lesions with specific and easily observable changes in morphology and color 9 18 , and substantiated its effectiveness through a prospective, multicenter, cross-sectional diagnostic study. This method detected a remarkable 90.9% of HGNs in a one-time screening session.

Ulcerative lesions are generally easy to detect. Biopsy is an accessible method to differentiate between neoplastic and non-neoplastic ulcerative lesions. Moreover, ulcer is one of the most important clues for gastric neoplastic lesions because ulcerative HGN accounts for 20% of all HGNs and nearly 10% of ulcerative lesions were neoplastic in our study (Supplementary Fig. 1 and Supplementary Table 1). In another previous study, about 70% EGC had endoscopic findings of ulceration 24 . Therefore, we choose ulceration as the first suspicious morphological characteristic for EGC.

In our previous study, we found that over 80% of HGNs originating from the esophagogastric junction (or cardia) demonstrated redness 18 . Another study by Goda et al. reported that nearly 90% of cardia EGCs were reddish on WLI 25 . Considering these findings, we propose that reddish lesions at the cardia area should increase the index of suspicion. On the other hand, most non-cardia HGNs outside the atrophic area are commonly associated with undifferentiated or mixed-type gastric cancers, which present as well-demarcated, or pale/faded under WLI. Such lesions demand special attention, particularly near the atrophic border. Thus, suspicious lesions outside the atrophic mucosa are classified into two types: reddish lesions localized at the EGJ/cardia and pale ones occurring in non-cardia regions. Incidence of *H. pylori-* naïve gastric cancer is relatively low 26 27 , and most *H. pylori-* naïve EGCs, including early signet-ring cell carcinoma and foveolar epithelial type, present as pale or well-demarcated lesions outside the atrophic area.

Lesions inside the atrophic area can be classified into elevated type and flat/depressed type. For elevated EGCs within the atrophic area, our previous studies 9 18 indicated that nearly 95% presented with clear borders or uneven tops. These two characteristics can effectively differentiate elevated EGCs from other lesions such as submucosal tumors or benign polyps. For flat/depressed lesions (gastritis-like lesions) within the atrophic area, Yao 4 and Muto 28 suggested that a well-demarcated border and irregular surface inferred diagnosis of EGC. Recent studies 20 29 have shown that EGC appears brownish or pink under NBI after eradication of *H. pylori* . Based on these reports, we chose irregular borders, uneven surface, and ocher (reddish-brown) appearance under NBI as three easily observable clues for flat/depressed EGC in the atrophic area. According to our previous study 18 , 90.8% of flat/depressed ECGs in the atrophic area presented with at least one of these three characteristics.

To determine whether the aforementioned endoscopic morphological features constitute independent risk factors for EGC, we performed multivariable regression analysis incorporating established gastric cancer risk factors 12 , including demographic characteristics, lifestyle factors, personal and family medical history, *H. pylori* infection, and gastric atrophy. Lesions exhibiting these morphological features demonstrated a 42-fold increased likelihood of EGC (adjusted OR 42.03, 95% CI 11.14–158.63).

The protocol missed five diagnoses among 55 HGN lesions. Of these, two were *H. pylori* negative, one occurred after *H. pylori* eradication, one involved active *H. pylori* infection, and the *H. pylori* status of the remaining case was unknown. In the two *H. pylori* negative cases, one lesion resembled a submucosal tumor located at the cardia and the other presented as spontaneous bleeding in the gastric body. The third miss was a lesion with unclear *H. pylori* status, described as an isochromatic depressed area located at the cardia; these lesions were biopsied based on endoscopist experience. Another missed case occurred after *H. pylori* eradication, located at the gastric angle, with gastritis-like features; biopsy was performed according to VS classification. The final missed case involved an active *H. pylori* infection, also presenting with gastritis-like features at the gastric antrum, detected by random biopsy. Among 931 lesions biopsied for suspicious morphological characteristics, 810 lesions were negative for neoplasia. The most common false-positive lesions were “elevated lesions with clear borders inside atrophic area” (131 cases), followed by “flat/depressed lesions with irregular borders inside atrophic area” (128 cases). Although we characterized these gastritis-like EGC features with as much detail as possible, the false-positive and false-negative cases remained focused on gastritis-like lesions.

Our study has certain limitations. First, as a cross-sectional study, the follow-up data were lacking, which means that actual prevalence of HGN remains unknown. Consequently, the sensitivity in this study might have been overestimated. Second, although our study demonstrated an impressive sensitivity of 90.9%, exceeding that of magnifying NBI (approximately 60%) from a different study 1 , or other non-magnifying methods like chromoendoscopy (approximately 78%) 30 , there were no direct comparisons between our method and the other methods. Moreover, the specificity (67.8%) and PPV (5.4%) of this study was notably lower compared with magnifying NBI 1 30 (94.3% and 57.1%), which may lead to increased pathological workload and potential costs. Further research is needed to directly compare our method with magnifying gastroscopy or evaluate the feasibility of combining the two methods.

## Conclusions

In conclusion, our study demonstrated that targeted biopsy of suspicious lesions with non-magnifying gastroscopy could be effective for detecting EGC based on several simple and easily observable changes in morphology and color. This method can be used as a primary clue for detecting EGC. In the future, studies that compare and combine this non-magnifying observation method with magnifying gastroscopy are warranted.

## Footnotes

* Shared last authorship.

## Supplementary Material

Supplementary Material Zusatzmaterial
