# Supplementary material for: Diagnostic value of non-magnifying gastroscopy followed by targeted biopsy for detection of early gastric cancer: Multicenter prospective study
Source: Endosc Int Open. 2026 Mar 16;14:a27816191. doi: 10.1055/a-2781-6191 (PMC13063301; doi:10.1055/a-2781-6191)
Supplement: Supplementary file 1 — Supplementary Material [file 10-1055-a-2781-6191_28038319.pdf]

Supplementary Table 1 Histology of lesions with various high-risk morphological characteristics.

	Total n	GEN n, % (95%CI)		HGN n, % (95%CI)	
**Ulcerative lesions**	123	14	11.4% (5.7%-17.1%)	11	8.9% (3.8%-14.1%)
**Outside the atrophic area**					
Reddish in esophagogastric junction	75	8	10.7% (3.5%-17.8%)	4	5.3% (0.1%-10.5%)
Clear borders	80	8	10.0% (3.3%-16.7%)	3	3.8% (0.5%-8.0%)
Pale	56	4	7.1% (0.2%-14.1%)	2	3.6% (1.4%-8.6%)
**Inside the atrophic area**					
Elevated with clear borders	148	11	7.4% (3.2%-11.7%)	7	4.7% (1.3%-8.2%)
Elevated with uneven top	53	9	17.0% (6.5%-27.4%)	5	9.4% (1.3%-17.6%)
Flat/depressed with irregular borders	151	17	11.3% (6.2%-16.4%)	12	7.9% (3.6%-12.3%)
Flat/depressed with uneven surface	124	6	4.8% (1.0%-8.7%)	5	4.0% (0.5%-7.5%)
Ochrous under NBI	84	10	11.9% (4.8%-19.0%)	5	6.0% (0.8%-11.1%)

GEN, gastric epithelial neoplasia; HGN, high-grade neoplasia; NBI, narrow-band imaging.

Supplementary Figure 1 Morphological characteristics of the HGNs.

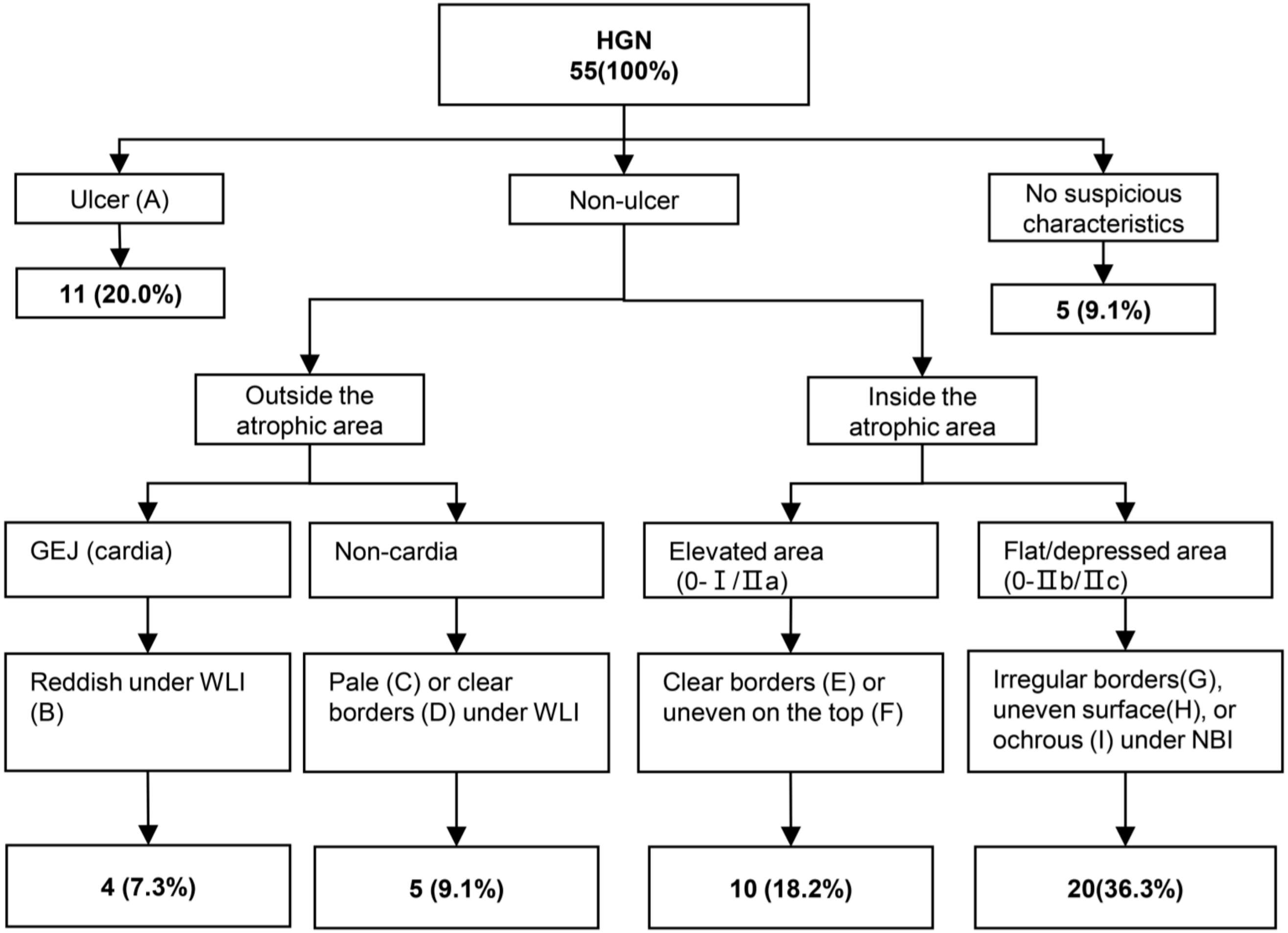

Endosc Int Open | DOI 10.1055/a-2781-6191 | © The Author(s).
